# A New Conformal Map for Polynomial Chaos Applied to Direction-of-Arrival Estimation via UCA Root-MUSIC

**DOI:** 10.3390/s22145229

**Published:** 2022-07-13

**Authors:** Seppe Van Brandt, Jo Verhaevert, Tanja Van Hecke, Hendrik Rogier

**Affiliations:** IDLab, Department of Information Technology, Faculty of Engineering and Architecture, Ghent University-imec, 9052 Gent, Belgium; jo.verhaevert@ugent.be (J.V.); tanja.vanhecke@ugent.be (T.V.H.); hendrik.rogier@ugent.be (H.R.)

**Keywords:** conformal map, polynomial chaos, root-MUSIC, direction-of-arrival, uniform circular array, deformation, error propagation

## Abstract

The effects of random array deformations on Direction-of-Arrival (DOA) estimation with root-Multiple Signal Classification for uniform circular arrays (UCA root-MUSIC) are characterized by a conformally mapped generalized Polynomial Chaos (gPC) algorithm. The studied random deformations of the array are elliptical and are described by different Beta distributions. To successfully capture the erratic deviations in DOA estimates that occur at larger deformations, specifically at the edges of the distributions, a novel conformal map is introduced, based on the hyperbolic tangent function. The application of this new map is compared to regular gPC and Monte Carlo sampling as a reference. A significant increase in convergence rate is observed. The numerical experiments show that the UCA root-MUSIC algorithm is robust to the considered array deformations, since the resulting errors on the DOA estimates are limited to only 2 to 3 degrees in most cases.

## 1. Introduction

Direction-of-arrival (DOA) estimation is a fundamental enabler of current and next-generation wireless communication systems. With the arrival of 5G and the development of 6G, it is of great importance to understand how DOA techniques perform under imperfect conditions, especially as the effects of hardware impairments become more important when the operating frequency increases [1]. Simulation times can be large and Monte Carlo (MC) analysis is often too time-consuming due to the excessive number of realizations that must be evaluated. Therefore, a fitting stochastic framework for the characterization of uncertainty propagation within these systems is Generalized Polynomial Chaos (gPC) [2,3]. As a versatile technique, gPC has been applied extensively to study the effects of randomness on antenna performance and radio wave propagation [4,5,6,7,8,9,10,11]. With the appropriate approach, gPC can even compete with MC when a high number of random variables are used [12,13,14,15,16,17]. In the context of localization, gPC has also been used—for example, in [18,19], where the effects of element displacements on DOA estimation were investigated; in [20], where the effects of random element gain and phase variations were studied, and in [21], where the uncertainty in multiple angle-of-arrival measurements was translated into an uncertainty in position estimation.

Being based on polynomials, however, gPC has its own drawbacks, one of the most important ones being slow convergence in the presence of function singularities [22,23]. Conformal maps can alleviate these problems, as demonstrated on Maxwell’s source problem in [24]. Conformal maps were used previously in [25] to compute more accurate quadrature rules for integrands with similar non-polynomial behavior, inspired by [26,27].

In this work, we extend the techniques described in [24] by introducing a novel map that compensates for (apparent) singularities on the real axis. We illustrate the effectiveness of this new conformal map by characterizing the effects of elliptical array deformations on root-Multiple Signal Classification for uniform circular arrays (UCA root-MUSIC) [28], as it is a well-established and efficient DOA estimation algorithm. The UCA topology is a relatively simple array geometry that enables DOA estimation of the azimuth angle over a full 360° field of view, besides some more limited capability to provide estimates of the elevation angle. However, the dedicated UCA-root-MUSIC algorithm heavily relies on the inherent circular symmetry of the antenna array. Therefore, this algorithm and array topology are the ideal candidates to study the effects of random deformations on DOA estimation techniques. To be concise and keep the focus on the presence of singularities, we do not include white noise, in contrast to [18,19,20].

In the next section, key concepts are introduced and the novel conformal map is revealed in the appropriate mathematical framework. In Section 3 and Section 4, the computational results are presented and discussed, and in the final section, Section 5, a conclusion is given.

## 2. Methods

### 2.1. gPC Approximation

Generalized Polynomial Chaos (gPC) [2,3] approximates a function *f* of a random variable *x* by an expansion in a well-chosen orthogonal polynomial basis $\left\{\Phi_{k}\right\}$. The orthogonal polynomials in question are associated with the weight function *w*, representing the probability density function of the random variable being used, typically according the Askey scheme [2]. If *f* is a function that is (computationally) expensive to evaluate, a gPC approximation of *f* can provide a computationally cheap substitute.

Assuming that *w* is a weight function with support [−1, 1], the gPC orthogonal projection of degree *N* is defined as

(1) $$P_{N}f\left(x\right)=\sum_{k=0}^{N}t_{k}\Phi_{k}\left(x\right),\;\;\;t_{k}=C_{k}^{-1}\cdot \int_{-1}^{1}f\left(x\right)\Phi_{k}\left(x\right)w\left(x\right)dx,$$

with $C_{k}=\int_{-1}^{1}\Phi_{k}^{2}\left(x\right)w\left(x\right)x$. Once the expansion coefficients $t_{k}$ are known, the first two moments of *f* can be calculated and estimated by

(2) $$\begin{array}{ccc} \mu & = & \int_{-1}^{1}f\left(x\right)w\left(x\right)dx=t_{0}\\ \mu_{2} & = & \int_{-1}^{1}f^{2}\left(x\right)w\left(x\right)dx\approx \sum_{k=0}^{N}t_{k}^{\;2}\cdot C_{k}, \end{array}$$

while the standard deviation on *f* can be estimated by

(3) $$\sigma =\sqrt{\mu_{2}-\mu^{2}}\approx {\left(\sum_{k=1}^{N}t_{k}^{\;2}\cdot C_{k}\right)}^{\frac{1}{2}}.$$

The error in the $L^{2}$-norm ${∥\cdot ∥}_{L^{2},w}$ can be written in terms of the standard deviation σ and its estimator:

(4) $$\begin{array}{cc} ∥f-P_{N}{f∥}_{L^{2},w} & ={\left(\int_{-1}^{1}{\left[f\left(x\right)-P_{N}f\left(x\right)\right]}^{2}w\left(x\right)dx\right)}^{\frac{1}{2}}\\ \; & ={\left(\int_{-1}^{1}{\left[\sum_{k=0}^{\infty }t_{k}\Phi_{k}\left(x\right)-\sum_{k=0}^{N}t_{k}\Phi_{k}\left(x\right)\right]}^{2}w\left(x\right)dx\right)}^{\frac{1}{2}}\\ \; & ={\left(\int_{-1}^{1}{\left[\sum_{k=N+1}^{\infty }t_{k}\Phi_{k}\left(x\right)\right]}^{2}w\left(x\right)dx\right)}^{\frac{1}{2}}={\left(\sum_{k=N+1}^{\infty }t_{k}^{2}\cdot C_{k}\right)}^{\frac{1}{2}}\\ \; & ={\left(\sum_{k=1}^{\infty }t_{k}^{2}\cdot C_{k}-\sum_{k=1}^{N}t_{k}^{2}\cdot C_{k}\right)}^{\frac{1}{2}}={\left(\sigma^{2}-\sum_{k=1}^{N}t_{k}^{\;2}\cdot C_{k}\right)}^{\frac{1}{2}}. \end{array}$$

**Figure 1:**
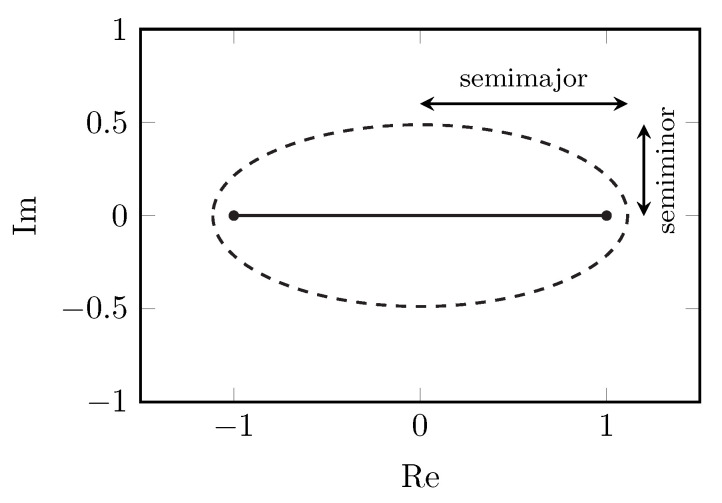
The $E_{1.6}$ Bernstein ellipse. Its size ρ is equal to the sum of its semiminor and semimajor axes—in this case, 1.6. Its foci −1 and 1 are shown by the black dots.

The quality of the gPC approximation will depend upon the analyticity of *f* in the complex plane [3,22,23]. The analyticity of *f* can be described by a Bernstein ellipse $E_{\rho }$, which is defined as the ellipse with foci −1 and 1 and ρ as the sum of its semiminor and semimajor axis; see Figure 1. From Bernstein’s theorem for the convergence of the Chebyshev projection $P_{N}^{\mathrm{Ch}}$ [22,23] and the fact that the gPC projection $P_{N}$ minimizes the $L^{2}$-error [3], it follows that, if *f* is analytically continuable to the open Bernstein ellipse $E_{\rho }\subseteq C$, the error on the *N*th degree gPC approximation, according to the $L^{2}$-norm, is limited to

(5) $$∥f-P_{N}{f∥}_{L^{2},w}\leq ∥f-P_{N}^{\mathrm{Ch}}{f∥}_{L^{2},w}\leq ∥f-P_{N}^{\mathrm{Ch}}{f∥}_{\infty }\cdot {∥1∥}_{L^{2},w}\leq 2\frac{M\rho^{-N}}{\rho -1},$$

with ${∥\cdot ∥}_{\infty }$ the supremum norm and |f(x)|≤M for $x\in E_{\rho }$. It is clear from Equation (5) that it is preferable to have a high ρ, i.e., a large Bernstein ellipse, for optimal convergence. Singularities in the complex plane, close to the interval of interest [−1, 1], can substantially lower ρ and will, therefore, be detrimental to the gPC approximation.

In most practical applications, the integral in Equation (1) is not analytically computable. In 1D cases, the coefficients $t_{k}$ are generally approximated numerically using a Gauss quadrature rule [3]:

(6) $$t_{k}\approx C_{k}^{-1}\cdot \sum_{i=1}^{N+1}f\left(x_{i}\right)\Phi_{k}\left(x_{i}\right)w_{i},$$

with $\left(x_{i},w_{i}\right)$ being the N+1 quadrature nodes and weights of polynomial accuracy 2N+1 associated with *w*. According to [3], Section 3.6, the aliasing error on the polynomial expansion of *f* is given by

(7) $$A_{N}f\left(x\right)=\sum_{j=N+1}^{\infty }t_{j}\sum_{k=0}^{N}C_{k}^{-1}\left[\sum_{i=1}^{N+1}\Phi_{j}\left(x_{i}\right)\Phi_{k}\left(x_{i}\right)w_{i}\right]\Phi_{k}\left(x\right).$$

In essence, the aliasing error follows from the fact that the lower-order polynomials $\left\{\Phi_{k}\right\}$ cannot be distinguished from the higher-order polynomials $\left\{\Phi_{j}\right\}$ on a finite grid. As seen in Equation (7), it can be interpreted as the error that is introduced by using the lower-order discrete expansion of the higher-order polynomials instead of the higher-order polynomials themselves. In [3], it is mentioned that the aliasing error induced by using Formula (6) is usually of the same order as the projection error in Equation (5). Hence, the aliasing error will also benefit from a higher ρ.

### 2.2. Conformally Mapped gPC

In [24], a framework was established to incorporate conformal maps into the gPC algorithm for enlarging the Bernstein ellipse, based on earlier research from [25]. Consider a map *g* that is conformal in an open region Ω⊆C with subdomain [−1, 1]⊆R, with g([−1, 1])=[−1, 1] and g(±1)=±1. This map can be used to define a new variable $\tilde{x}$:

(8) $$\left\{\begin{array}{c} x=g(\tilde{x})\\ \tilde{x}=g^{-1}\left(x\right). \end{array}\right.$$

If *x* is a random variable, distributed according to the weight function w(x) with support [−1, 1], the variable after transformation $\tilde{x}$ will have its own weight function $\tilde{w}\left(\tilde{x}\right)$ with support [−1, 1] [24]:

$$\begin{array}{cc} \tilde{w}\left(\tilde{x}\right) & =\left[w\circ g\right]\left(\tilde{x}\right)\left|\frac{dx}{d\tilde{x}}\right|\\ \; & =\left[w\circ g\right]\left(\tilde{x}\right)\left|g^{\prime }\left(\tilde{x}\right)\right|. \end{array}$$

The symbol ∘ is used to denote function composition, i.e., $\left[f_{1}\circ f_{2}\right]\left(x\right)=f_{1}\left(f_{2}\left(x\right)\right)$. By choosing $g^{\prime }>0$ in Ω, the above equation becomes

(9) $$\tilde{w}\left(\tilde{x}\right)=\left[w\circ g\right]\left(\tilde{x}\right)g^{\prime }\left(\tilde{x}\right).$$

Assuming that the orthogonal polynomials $\left\{\Phi_{k}\right\}$ associated with *w* are known, the orthogonal polynomials $\left\{{\tilde{\Phi }}_{k}\right\}$ associated with $\tilde{w}$ can be constructed by the Modified Chebyshev Algorithm [29]. Next, the quadrature points ${\tilde{x}}_{i}$ and associated weights ${\tilde{w}}_{i}$ of $\tilde{w}$ can be calculated using the Golub–Welsch Algorithm [30]. The Modified Chebyshev Algorithm is based upon a set of integrals called the modified moments, defined by

(10) $$m_{k}=\int_{-1}^{1}\Phi_{k}\left(\tilde{x}\right)\tilde{w}\left(\tilde{x}\right)d\tilde{x}.$$

Only for select combinations of weight function and conformal map can these integrals be calculated analytically. In other cases, one has to make use of quadrature rules (or other computational methods) to evaluate these integrals numerically. As the quadrature nodes and weights belonging to $\tilde{w}$ are not yet known at this point in the algorithm, this integral has to be reformulated as an integral with weight function *w*, whose quadrature nodes and weights are known. One means of achieving this is by rewriting the integral as

(11) $$\begin{array}{cc} m_{k} & =\int_{-1}^{1}\Phi_{k}\left(\tilde{x}\right)\tilde{w}\left(\tilde{x}\right)d\tilde{x}\\ \; & =\int_{-1}^{1}\Phi_{k}\left(x\right)\frac{\tilde{w}\left(x\right)}{w(x)}w\left(x\right)dx\\ \; & \approx \sum_{i=1}^{N_{m}}\Phi_{k}\left(x_{i}\right)\frac{\tilde{w}\left(x_{i}\right)}{w(x_{i})}\cdot w_{i}. \end{array}$$

with $\left(x_{i},w_{i}\right)$ the quadrature nodes and weights of polynomial accuracy $2N_{m}-1$ associated with *w*. Within this research, the value of $N_{m}$ was set to 50, a value for which sufficient accuracy was reached.

Instead of a polynomial expansion of *f*, in the conformally mapped gPC algorithm introduced by [24], an expansion of f∘g is performed, using the newly constructed ${\tilde{\Phi }}_{k}$ polynomials:

(12) $$P_{N}\left[f\circ g\right]\left(\tilde{x}\right)=\sum_{k=0}^{N}{\tilde{t}}_{k}{\tilde{\Phi }}_{k}\left(\tilde{x}\right).$$

By substituting the inverse map $\tilde{x}=g^{-1}\left(x\right)$ at both sides of Equation (12), a mapped approximation of *f* is obtained:

(13) $${\tilde{P}}_{N}f\left(x\right)=\sum_{k=0}^{N}{\tilde{t}}_{k}\left[{\tilde{\Phi }}_{k}\circ g^{-1}\right]\left(x\right).$$

**Scheme 1:**
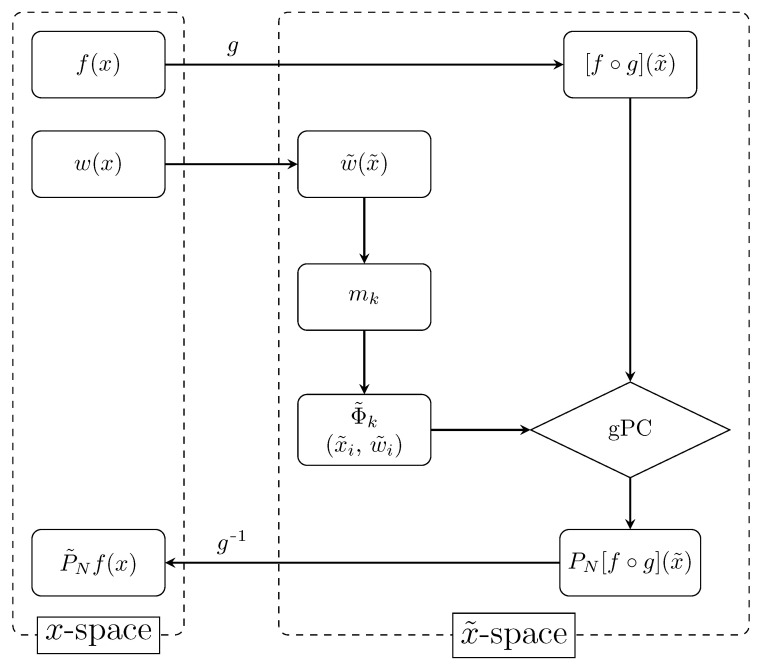
An overview of the conformally mapped gPC algorithm. A new random variable $\tilde{x}$ is defined by applying a conformal map $x=g(\tilde{x})$ on the random variable *x*. An adapted quadrature rule $\left({\tilde{x}}_{i},{\tilde{w}}_{i}\right)$ is constructed for the new weight function $\tilde{w}\left(\tilde{x}\right)$ via the modified moments $m_{k}$, along with its own orthogonal polynomials $\left\{{\tilde{\Phi }}_{k}\right\}$. Generalized Polynomial Chaos (gPC) is applied to the composite function $\left[f\circ g\right]$ and a mapped gPC approximation of *f* is found after performing the inverse conformal map $\tilde{x}=g^{-1}\left(x\right)$.

A schematic overview of this algorithm is shown in Scheme 1.

Analogously to classic gPC, the coefficients ${\tilde{t}}_{k}$ can be approximated by means of discrete projection, yielding

(14) $$\begin{array}{cc} {\tilde{t}}_{k} & ={\tilde{C}}_{k}^{-1}\cdot \int_{-1}^{1}\left[f\circ g\right]\left(\tilde{x}\right){\tilde{\Phi }}_{k}\left(\tilde{x}\right)\tilde{w}\left(\tilde{x}\right)d\tilde{x}\\ \; & \approx {\tilde{C}}_{k}^{-1}\cdot \sum_{i=1}^{N+1}\left[f\circ g\right]\left({\tilde{x}}_{i}\right){\tilde{\Phi }}_{k}\left({\tilde{x}}_{i}\right){\tilde{w}}_{i}, \end{array}$$

with ${\tilde{C}}_{k}=\int_{-1}^{1}{\tilde{\Phi }}_{k}^{2}\left(\tilde{x}\right)\tilde{w}\left(\tilde{x}\right)d\tilde{x}$ and $\left({\tilde{x}}_{i},{\tilde{w}}_{i}\right)$ the quadrature nodes and weights of polynomial accuracy 2N+1 associated with $\tilde{w}$.

An upper bound similar to the one in Equation (5) can be derived for the conformally mapped gPC expansion [3,22,23,24]:

(15) $$∥f-{\tilde{P}}_{N}{f∥}_{L^{2},w}={∥f\circ g-P_{N}\left[f\circ g\right]∥}_{L^{2},\tilde{w}}\leq 2\frac{\tilde{M}{\tilde{\rho }}^{-N}}{\tilde{\rho }-1}.$$

In this case, $\tilde{\rho }$ is the size of the open Bernstein ellipse $E_{\tilde{\rho }}$, in which f∘g is analytically continuable. It is apparent from Equations (5) and (15) that the conformal map *g* needs to be chosen in such a way that $\tilde{\rho }$ exceeds ρ, thus achieving a faster convergence. In other words, f∘g needs to be analytically continuable in a larger open Bernstein ellipse than *f*.

**Figure 2:**
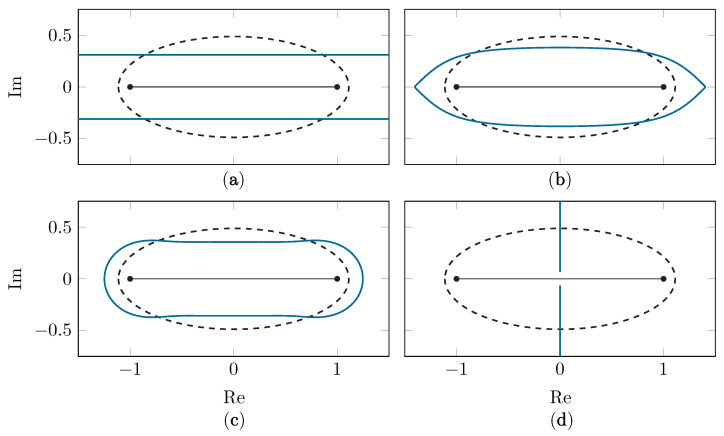
The $E_{1.6}$ Bernstein ellipse (dashed black line) and its image (solid blue line) under the Ellipse-to-Strip map (**a**), the KTE map (**b**), the Sausage map (**c**) and the Ellipse-to-Slit map with slits along the imaginary axis (**d**).

To the best of the authors’ knowledge, only one class of conformal map has been applied in the context of polynomial-based methods. This set of maps shifts singularities directly above or below the [−1, 1] interval, away from the origin, in order to achieve a larger Bernstein ellipse. The Ellipse-to-Strip map [25], the Sausage map [25], the Kosloff Tal-Ezer (KTE) map [27] and the Ellipse-to-Slit map [31] all fall into this category. In Figure 2, examples are shown of these established maps.

However, it is possible to encounter function singularities in other locations of the complex plane, such as on the real axis, close to the [−1, 1] interval. In these situations, there is a need for a new conformal map, which is proposed further in Section 2.3.

### 2.3. Tanh Map

Assume that *f* has a singularity on the real axis at location *p* with |p|>1. Due to this singularity, the size of the Bernstein ellipse $E_{\rho }$ associated with *f* (in the *x*-space) is limited to $\rho =\left|p\right|+\sqrt{p^{2}-1}$. Ideally, one would use a map that gives rise to a Bernstein ellipse $E_{\tilde{\rho }}$, associated with f∘g in the $\tilde{x}$-space, that is larger than $E_{\rho }$. It is clear that a suitable map for this purpose should shift the singularity away from the [−1, 1] interval. We propose the *tanh* map for this purpose:

(16) $$x=g\left(\tilde{x};\kappa \right)=\frac{\tanh(\kappa \tilde{x})}{\tanh(\kappa )}.$$

**Figure 3:**
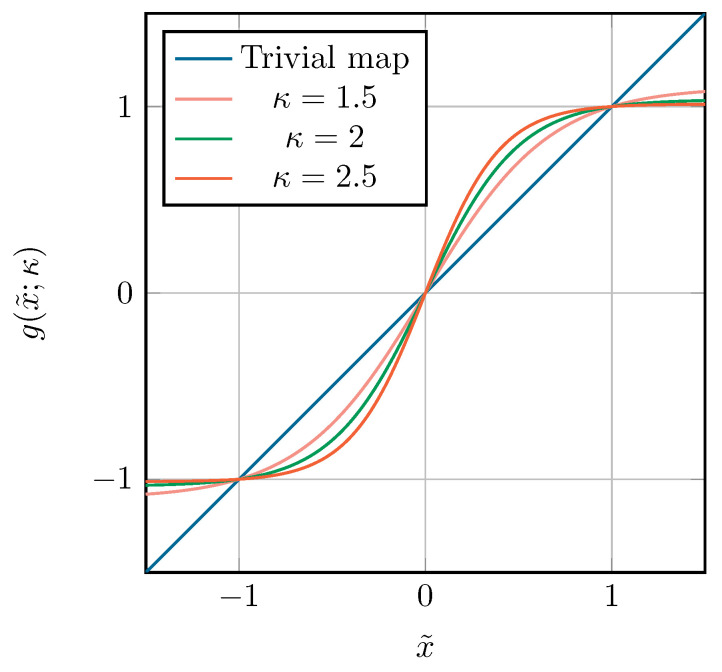
The *tanh* map for real $\tilde{x}$ at different values of κ, together with the trivial map $g\left(\tilde{x}\right)=\tilde{x}$.

An illustration of this map for a real *x* and $\tilde{x}$, and for different values of κ, is shown in Figure 3. The parameter κ is added to make the map adaptable to different values of the singularity’s location *p*.

The tanh(z) function is periodic with period πj, i.e., tanh(z)=tanh(z±πj), and has simple poles at $\frac{\pi }{2}j\pm \pi jl$, with l∈Z. As the map needs to be bijective in order to define the inverse map, the domain of the above map is restricted to the strip around the real axis between its closest poles:

(17) |Im(x˜)|<π2κj.

The inverse of Equation (16) can now be defined as

(18) $$\tilde{x}=g^{-1}\left(x;\kappa \right)=\frac{1}{2\kappa }\ln\left(\frac{1+x\tanh(\kappa )}{1-x\tanh(\kappa )}\right).$$

Using the *tanh* map, the singularity will shift to a position $|\tilde{p}\left|=\right|g^{-1}\left(p;\kappa \right)|>|p|$. As $g^{-1}\left(p;\kappa \right)$ needs to be defined and $g^{-1}\left(x;\kappa \right)$ has branch cuts $]-\infty ,-\frac{1}{\tanh(\kappa )}]$ and $[\frac{1}{\tanh(\kappa )},+\infty [$, κ is fundamentally limited to

(19) $$\kappa <\kappa_{\max}=\ln\left(\frac{|p|+1}{|p|-1}\right).$$

Two Bernstein ellipses in the $\tilde{x}$-space can be defined: one that only takes into account the shifted singularity $\tilde{p}$ with size

(20) $${\tilde{\rho }}_{\mathrm{re}}=\left|\tilde{p}\right|+\sqrt{{\tilde{p}}^{2}-1}=\frac{1}{2\kappa }\left|\ln\left(\frac{1+p\tanh(\kappa )}{1-p\tanh(\kappa )}\right)\right|+\sqrt{\frac{1}{4\kappa^{2}}\ln{\left(\frac{1+p\tanh(\kappa )}{1-p\tanh(\kappa )}\right)}^{2}-1},$$

and one ellipse that only takes into account the singularities $\pm \frac{\pi }{2\kappa }j$ introduced by the *tanh* map, with size

(21) $${\tilde{\rho }}_{\mathrm{im}}=\frac{\pi }{2\kappa }+\sqrt{\frac{\pi^{2}}{4\kappa^{2}}+1}.$$

**Figure 4:**
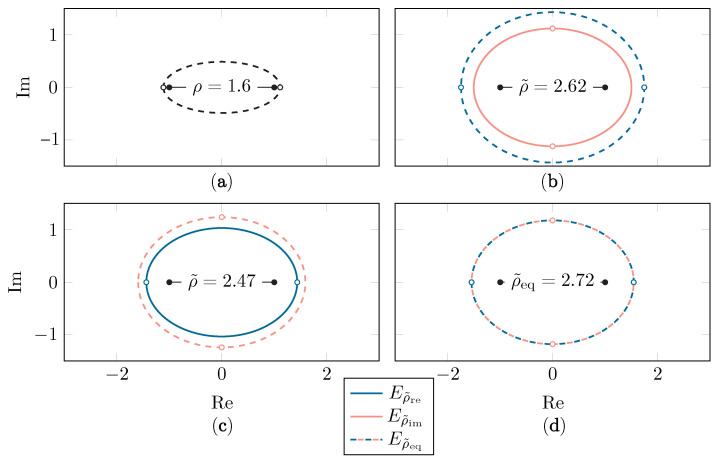
The Bernstein ellipse with size ρ=1.6 (singularity located at p=±1.1125) in the *x*-space (**a**) and the corresponding Bernstein ellipses $E_{{\tilde{\rho }}_{\mathrm{re}}}$ and $E_{{\tilde{\rho }}_{\mathrm{im}}}$ when using $\kappa =1.05\kappa_{\mathrm{eq}}$ (**b**), $\kappa =0.95\kappa_{\mathrm{eq}}$ (**c**) and $\kappa =\kappa_{\mathrm{eq}}$ (**d**). The singularities are depicted with hollow dots.

**Figure 5:**
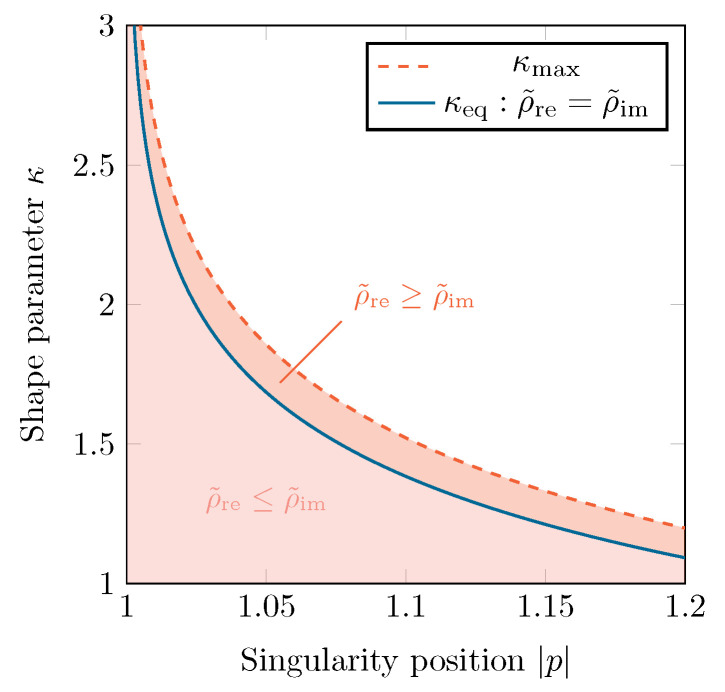
The different regions, denoting the relative sizes of both Bernstein ellipses, in the (|p|,κ)-space.

Depending on the values of *p* and κ, either $E_{{\tilde{\rho }}_{\mathrm{re}}}$ or $E_{{\tilde{\rho }}_{\mathrm{im}}}$ will be the largest ellipse. As the presence of singularities is restrictive for the gPC algorithm, $\tilde{\rho }$ is equal to the size of the smallest of both ellipses, i.e., $\tilde{\rho }=\min\left({\tilde{\rho }}_{\mathrm{re}},{\tilde{\rho }}_{\mathrm{im}}\right)$. The largest and optimal value of $\tilde{\rho }$, named ${\tilde{\rho }}_{\mathrm{eq}}$, is reached when both ellipses overlap $\left({\tilde{\rho }}_{\mathrm{re}}={\tilde{\rho }}_{\mathrm{im}}\right)$ at a certain $\kappa_{\mathrm{eq}}$, which can be found by solving Equations (20) and (21). Figure 4 illustrates this procedure for a singularity located at p=±1.1125. The value of $\kappa_{\mathrm{eq}}$ as a function of the singularity position |p| is shown in Figure 5, along with the two regions in (|p|,κ)-space, one where ${\tilde{\rho }}_{\mathrm{re}}\leq {\tilde{\rho }}_{\mathrm{im}}$ and one where ${\tilde{\rho }}_{\mathrm{re}}\geq {\tilde{\rho }}_{\mathrm{im}}$.

**Figure 6:**
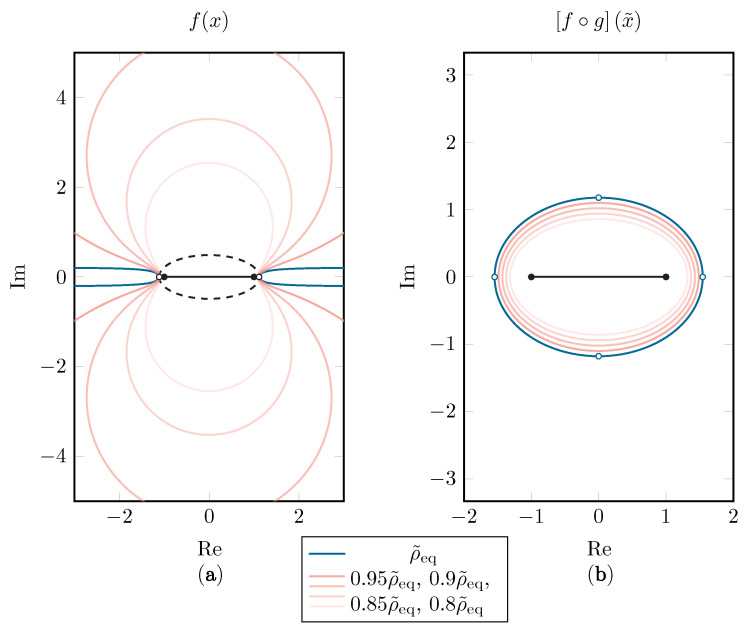
Starting with a Bernstein ellipse $E_{1.6}$ in the *x*-space, the Bernstein ellipses in the $\tilde{x}$-space with sizes ${\tilde{\rho }}_{\mathrm{eq}}=2.72$, $0.95{\tilde{\rho }}_{\mathrm{eq}}=2.59$, $0.9{\tilde{\rho }}_{\mathrm{eq}}=2.45$, $0.85{\tilde{\rho }}_{\mathrm{eq}}=2.31$ and $0.8{\tilde{\rho }}_{\mathrm{eq}}=2.18$ are shown (**b**). The corresponding regions $g(E_{\tilde{\rho }};\kappa_{\mathrm{eq}})$ in which analytic continuability of *f* is assumed are shown in (**a**). The original Bernstein ellipse $E_{1.6}$ is shown with a dashed line as a reference and the singularities are depicted with hollow dots.

**Figure 7:**
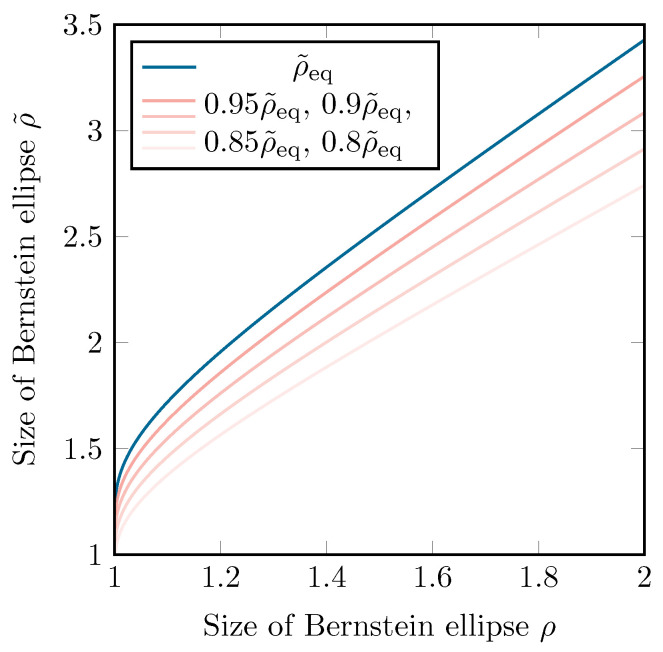
The size of $E_{\tilde{\rho }}$ as a function of the size of $E_{\rho }$ when using the *tanh* map. ${\tilde{\rho }}_{\mathrm{eq}}$ corresponds to the maximally achievable value of $\tilde{\rho }$.

Since $E_{{\tilde{\rho }}_{\mathrm{eq}}}$ has the singularities of *g*, being $\pm \frac{\pi }{2\kappa }j$, on its border, it will map to an infinitely large region $g(E_{{\tilde{\rho }}_{\mathrm{eq}}};\kappa_{\mathrm{eq}})$ in the *x*-space, as can be seen in Figure 6. This is similar to the Ellipse-to-Strip [25] and the Ellipse-to-Slit maps [31]. The condition that *f* is analytically continuable within this infinite region is rather strict, and if this is not the case, the effective Bernstein ellipse will be smaller than $E_{{\tilde{\rho }}_{\mathrm{eq}}}$. Luckily, the region of analyticity for *f* will shrink very quickly in comparison to the corresponding Bernstein ellipse $E_{\tilde{\rho }}$. Additionally, when comparing $\tilde{\rho }$ with ρ in Figure 7, one can conclude that, in practice, as long as singularities above and below the [−1, 1] interval are reasonably far away, the convergence rate gain does not suffer much when applying the *tanh* map.

### 2.4. Setup

**Figure 8:**
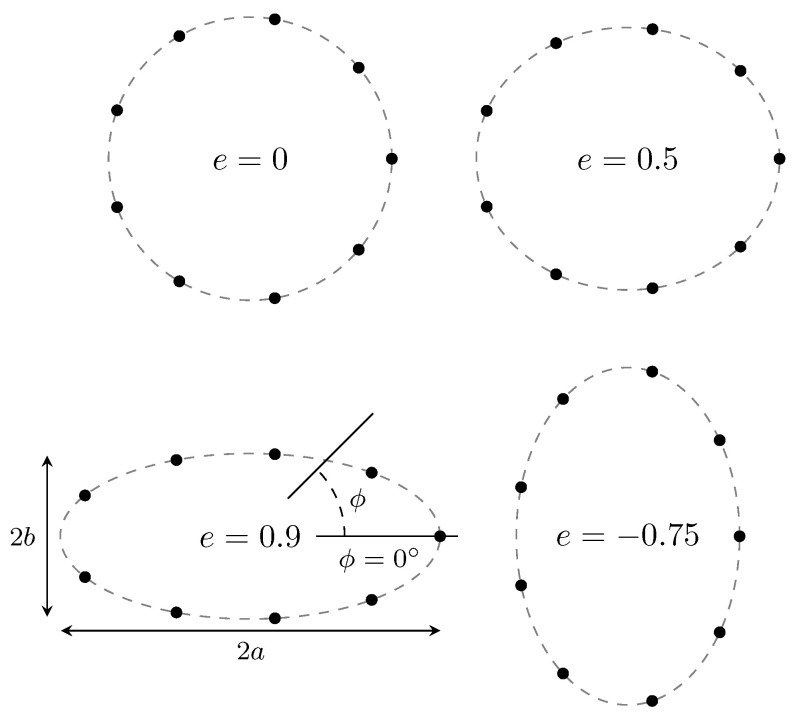
The shape of the deformed UCAs for different values of *e*.

Consider a nine-element uniform circular array (UCA) deployed in the azimuth plane. As the effects of the displacement of individual antenna elements on root-MUSIC have already been studied in earlier research—for example, in [18,19]—to showcase the proposed method, we assume that the array deforms in the shape of an ellipse with constant circumference and constant distance between the elements along the elliptical arc. This deformation is characterized by a single (random) variable: an “extended” eccentricity *e* defined by

(22) $$e=\left\{\begin{array}{cc} \sqrt{1-\frac{b}{a}} & \;\mathrm{if}\;a>b\\ -\sqrt{1-\frac{a}{b}} & \;\mathrm{if}\;a<b\\ 0 & \;\mathrm{if}\;a=b, \end{array}\right.$$

with 2a being the width of the array (along ϕ=0°) and 2b the height (along ϕ=90°). Different array deformations are illustrated in Figure 8.

We limit the eccentricity to the interval $\left[-0.9,\;0.9\right]$, since values outside of this interval were deemed too unrealistic and the deformation would become too large for the DOA algorithm to provide a DOA estimation. However, it is standard practice [24,25] to rescale random variables to $\left[-1,\;1\right]$. Therefore, the random variable *x* is introduced as e=0.9·x. We assume *x* to be distributed according to a Beta distribution [32]:

(23) $$\mathrm{Beta}\left(x;\alpha ,\beta \right)=\frac{{\left(1+x\right)}^{\alpha -1}{\left(1-x\right)}^{\beta -1}}{2^{\alpha +\beta -1}}\cdot \frac{\Gamma (\alpha +\beta )}{\Gamma (\alpha )\Gamma (\beta )}.$$

**Figure 9:**
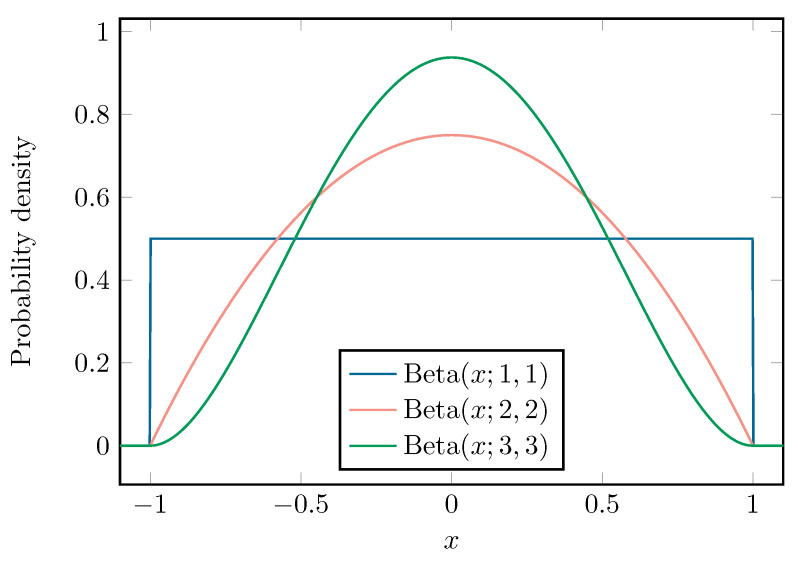
The three different Beta distributions used in this work.

Figure 9 illustrates three Beta distributions with shape parameters α=β=1, α=β=2 and α=β=3. The orthogonal polynomials $\left\{\Phi_{k}\right\}$ associated with the Beta distribution are the Jacobi polynomials [33].

Since the UCA root-MUSIC algorithm was calibrated with a circular array in mind, as the array deforms, the estimated DOAs will deviate from their correct positions. This error propagation is described by a function $\hat{\phi }=f\left(x\right)$, with $\hat{\phi }$ being the DOA estimation corresponding to one specific source.

From a practical viewpoint, it is non-intuitive to consider the analytic continuation of f(x) in the complex plane beyond the $\left[-1,\;1\right]$ interval. However, one can state that *f*, the relation between deformation and (erroneous) DOA estimation, behaves in a highly erratic manner when *x* approaches the edges of the interval $\left[-1,\;1\right]$. This behavior is equivalent to the presence of nearby singularities on the real axis. Therefore, the principles of Section 2.1, Section 2.2 and Section 2.3 apply to this case, even though f(x) has no closed form and the analytic continuation is not known.

## 3. Results

The operating frequency of the considered antenna array is set to 3.5 GHz (wavelength λ≈8.57cm), being the center of a 5G band [34]. The dipole elements are all of length λ/2, as is the radius of the UCA. The array is excited by six plane waves along directions ϕ=50°, 70°, 165°, 220°, 305° and 350° in the azimuth plane. DOA estimation is performed with the UCA root-MUSIC algorithm [28]. The full-wave NEC2++ simulator [35] is applied to rigorously simulate the complete antenna array, including all mutual coupling effects. For conciseness, we only discuss the behavior of one of the six DOA estimates, being the one corresponding to the source at ϕ=50°.

**Figure 10:**
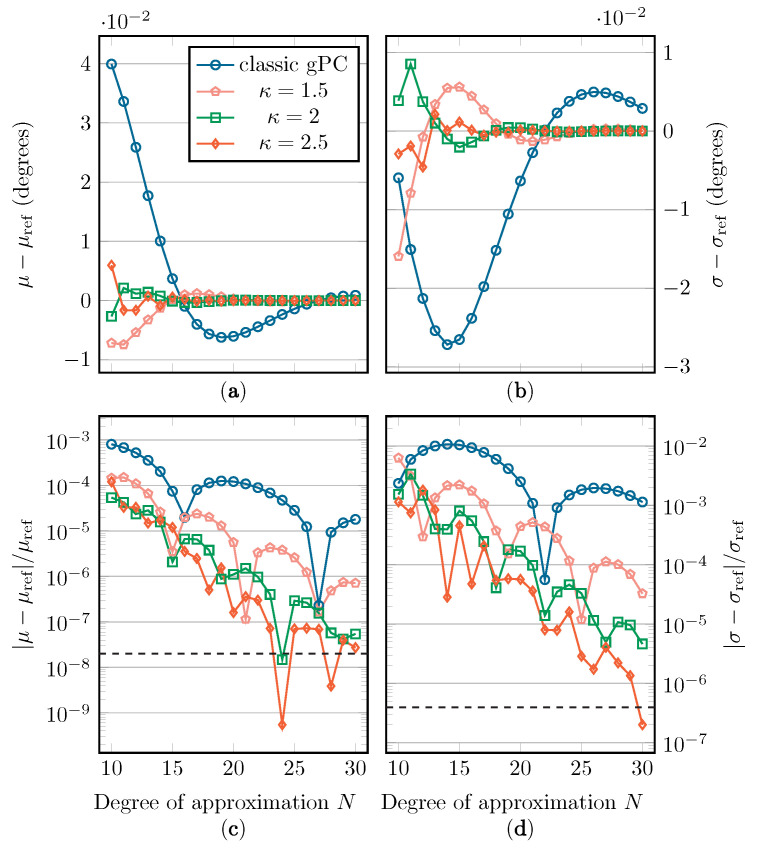
The absolute and relative errors on μ (**a**,**c**) and σ (**b**,**d**) with regard to their reference value when applying the Beta(x;1,1) distribution. The precision floor is shown by a dashed line.

**Figure 11:**
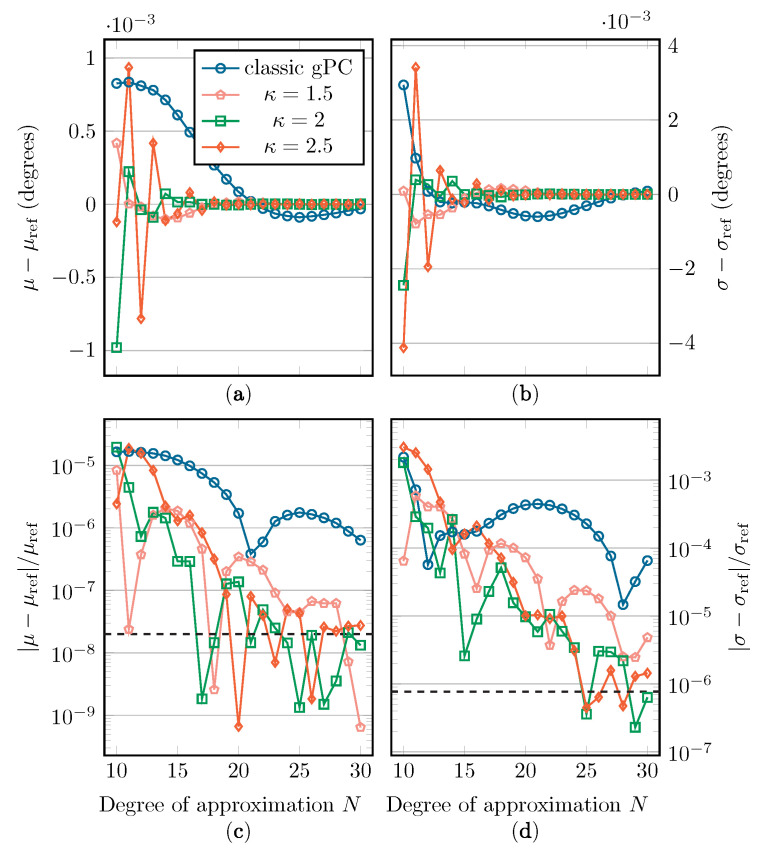
The absolute and relative errors on μ (**a**,**c**) and σ (**b**,**d**) with regard to their reference value when applying the Beta(x;2,2) distribution. The precision floor is shown by a dashed line.

**Figure 12:**
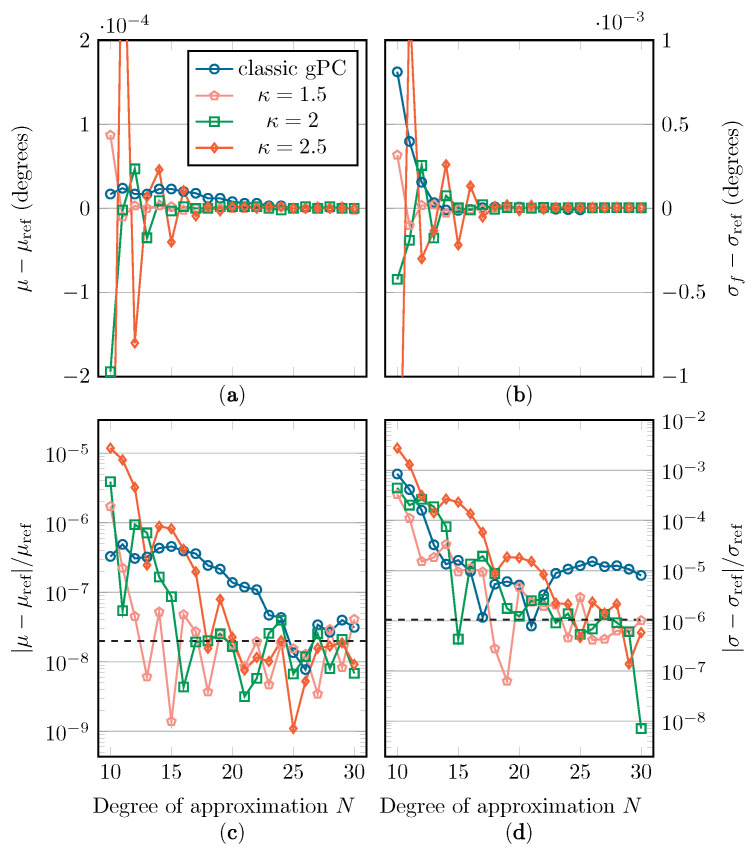
The absolute and relative errors on μ (**a**,**c**) and σ (**b**,**d**) with regard to their reference value when applying the Beta(x;3,3) distribution. The precision floor is shown by a dashed line.

In Figure 10, Figure 11 and Figure 12, the absolute and relative errors of μ and σ are presented as a function of *N* for the different Beta distributions. These values are calculated with classic gPC and mapped gPC for varying values of κ. The expansion coefficients are approximated with discrete projection according to Equations (6) and (14). As μ is equal to $t_{0}$ and ${\tilde{t}}_{0}$, the error shown in subfigures (c) is only the aliasing error introduced by the discrete projection. The error on the estimation of σ in subfigures (d) can, in addition to the aliasing error, be directly linked to the $L^{2}$-error according to Equation (4). As there are no analytical solutions to compare the results with, reference values are computed numerically by means of Monte Carlo simulation, using Latin Hypercube Sampling (LHS) with $10^{6}$ samples [36]. These values are displayed in Table 1. The implemented UCA root-MUSIC algorithm has a precision of around $10^{-6}$ degrees, which is why, in some graphs, convergence halts at a relative error of around $2\times 10^{-8}$ and $10^{-6}$ for μ and σ, respectively.

**Figure 13:**
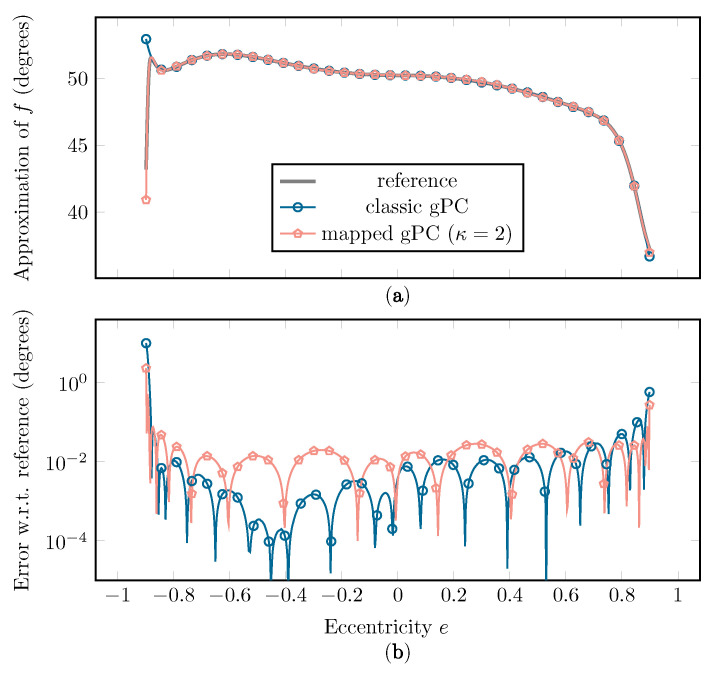
(**a**) The approximation of *f* using classic and mapped gPC and (**b**) the error on the approximation with regard to the reference curve, with w(x)=Beta(x;2,2) and N=15. The reference curve was constructed by sampling the full simulation.

**Figure 14:**
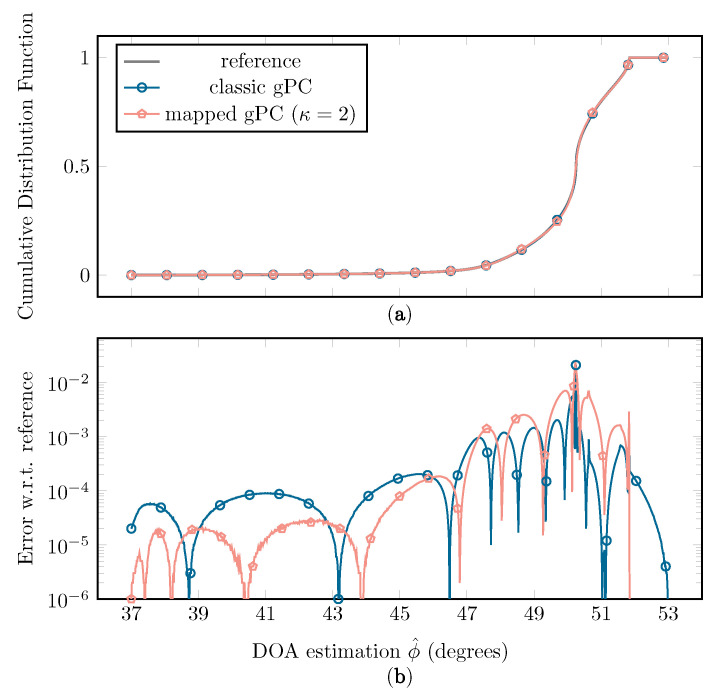
(**a**) The empirical CDFs of $\hat{\phi }$ when sampling the classic and mapped gPC expansion with the full simulation as a reference, each constructed with LHS and $10^{6}$ samples, with w(x)=Beta(x;2,2) and N=15. (**b**) The error of the classic and mapped gPC CDFs in comparison to the reference CDF.

In Figure 13, a comparison between the resulting classic and mapped gPC approximations of *f*, using the Beta(x;2,2) distribution and N=15, is shown. In Figure 14, the comparison of the resulting empirical cumulative distribution functions (CDFs) is plotted.

## 4. Discussion

The discussion is presented in two parts. First, the advantages of the use of the *tanh* map are analyzed, based on the deformed UCA application. Afterwards, the effects of the random array deformations on the UCA root-MUSIC algorithm are discussed.

### 4.1. Comparison of Classic and Mapped gPC

In Figure 10, Figure 11 and Figure 12, we see a general improvement when using mapped gPC over classic gPC, as, in all cases, mapped gPC reaches the precision bound the fastest. Comparing the results for the estimation of μ, in subfigures (a) and (c), in which only the aliasing error is present, we can confirm that the aliasing error does indeed benefit from an increase in the size of the Bernstein ellipse. As expected, this increase in convergence rate is also present in the results for the estimation of σ in subfigures (b) and (d), which affirms the principles from Section 2.2 and Section 2.3. Note that the error on σ is linked to the $L^{2}$-error in Equations (5) and (15) via Equation (4).

One aspect that should be mentioned is that, according to Equations (5) and (15), maximizing the size of the Bernstein ellipse only maximizes the rate of convergence, i.e., the incline of the convergence curves in subfigures (c) and (d). The vertical position of the convergence curves will, however, depend on other factors besides the size of the Bernstein ellipse. Therefore, it is possible that the fastest-converging method is not the one with the smallest error, especially in the cases with Beta(x;2,2) and Beta(x;3,3) as weight functions, where the precision floor is reached relatively quickly.

Two factors influence this phenomenon: first, the *M*/$\tilde{M}$ parameter in Equations (5) and (15), which is an upper bound of |f| in its supposed region of analyticity, being either $E_{\rho }$ for classic gPC or $g(E_{\tilde{\rho }};\kappa )$ for mapped gPC. Although it is difficult to establish closed-form mathematical relations for the value of *M*/$\tilde{M}$, for the mapped gPC case, one can state that an increase in κ will cause $\tilde{M}$ to either increase or stay the same, as clarified in Equation (24). In other words, an increase in convergence rate due to an increase in κ can be paired with an upward vertical shift in the convergence curve.

(24) $$\kappa_{1}<\kappa_{2}\Rightarrow g\left(E_{\tilde{\rho }};\kappa_{1}\right)\subset g\left(E_{\tilde{\rho }};\kappa_{2}\right)\Rightarrow {\tilde{M}}_{1}\leq {\tilde{M}}_{2}$$

Another factor is the aliasing error, defined by Equation (7), which is a function of the used weight function *w*/$\tilde{w}$ and the expansion polynomials $\left\{\Phi_{k}\right\}$/$\left\{{\tilde{\Phi }}_{k}\right\}$, adding a dependency on *w* and κ. An exact evaluation of Equation (7) is difficult. Upper bounds to the aliasing error are available for, among others, the Legendre and Chebychev polynomial expansions [37]; however, these are not readily applicable to this mapped gPC context. The dependency of both these factors on κ and *w* explains why we see different performance for the different κ values as the weight function changes, even though *f* and its singularities, and therefore also $\kappa_{\mathrm{eq}}$, stay the same. Unfortunately, it is difficult to establish closed-form mathematical relations for these dependencies.

Figure 13 shows that a better fit is achieved at the extremities of the interval when using mapped instead of classic gPC, resulting in a lower supremum error and $L_{2}$-error. As this erratic behavior of *f* in these regions has a large influence on the statistical moments of the function, a better fit at the edges will have a significant impact on the accuracy of the estimation of μ and σ. Another benefit of the mapped approach is a better approximation of the CDF at the far left and far right sides, as seen in Figure 14.

### 4.2. Consequences for the UCA Root-MUSIC Algorithm

In the situations studied in this paper, the introduction of a random deformation of the array causes a bias in the DOA estimator of 0.05 to 0.5 degrees and a standard deviation of the DOA estimation of 1 to 2.5 degrees (depending on the distribution shape; see Table 1). All things considered, this makes the UCA root-MUSIC algorithm rather robust against the array deformations studied in this work.

## 5. Conclusions and Future Work

The non-polynomial behavior of the root-MUSIC DOA estimation as a function of the elliptical deformation of the UCA can be compared to the presence of singularities on the real axis, close to the interval of interest, which have a detrimental effect on the convergence of the classic gPC algorithm. Luckily, using the newly defined *tanh* conformal map, these singularities are moved further away from the domain of the random variable, which makes for a better characterization of the erratic behavior of the DOA estimation and, as a result, causes a considerable increase in the convergence rate of the first- and second-order statistics in comparison to classic gPC.

We conclude from the simulations that the errors induced in the DOA estimation due to the elliptical deformation of the UCA are limited to only a few degrees in most cases. However, when the eccentricity reaches an absolute value of around 0.7, the DOA estimations become very volatile, with much larger errors of up to 10 degrees.

In future work, it can be of interest to develop and research even more specific conformal maps so that each type of function singularity can be dealt with in an efficient manner. As for the DOA estimation in 5G and 6G wireless communication networks, it might be advisable to look at other types of antenna arrays, such as rectangular arrays, which are currently integrated into 5G base stations and handsets [38]. Additionally, it could be interesting to look at other, modified types of MUSIC, such as or spatial/backward/time smoothing MUSIC, which are better equipped to deal with highly correlated signals, as encountered due to multipath propagation in indoor environments [39]. The technique could also be extended to hybrid techniques, such as SpotFi, that rely on a combination of time-of-flight and angle-of-arrival with MUSIC to perform accurate localization with common WiFi infrastructures [40].

## Figures and Tables

**Table 1:** The reference values of μ and σ. Computed with MC using LHS and $10^{6}$ samples.

	$\mu_{\mathrm{ref}}$	$\sigma_{\mathrm{ref}}$
Beta(x;1,1)	49.510102	2.527614
Beta(x;2,2)	50.046675	1.347420
Beta(x;3,3)	50.163825	0.956752

## Data Availability

Not applicable.
